# Prevention of mastitis in multiparous dairy cows with a previous history of mastitis by oral feeding with probiotic *Bacillus subtilis*

**DOI:** 10.1111/asj.13764

**Published:** 2022-09-09

**Authors:** Megumi Urakawa, Tao Zhuang, Hidetoshi Sato, Satoru Takanashi, Kozue Yoshimura, Yuma Endo, Teppei Katsura, Tsuyoshi Umino, Koutaro Tanaka, Hitoshi Watanabe, Hiroko Kobayashi, Naokazu Takada, Tomoyuki Kozutsumi, Hiroaki Kumagai, Takafumi Asano, Kohko Sazawa, Nobuhisa Ashida, Guoqi Zhao, Michael T. Rose, Haruki Kitazawa, Hitoshi Shirakawa, Kouichi Watanabe, Tomonori Nochi, Takehiko Nakamura, Hisashi Aso

**Affiliations:** ^1^ International Education and Research Center for Food and Agricultural Immunology, Graduate School of Agricultural Science Tohoku University Sendai Japan; ^2^ Laboratory of Animal Health Science, Graduate School of Agricultural Science Tohoku University Sendai Japan; ^3^ Laboratory of Animal Functional Morphology, Graduate School of Agricultural Science Tohoku University Sendai Japan; ^4^ Miyagi Prefectural Livestock Experiment Station Osaki Japan; ^5^ Institute of Animal Culture Collection and Application, College of Animal Science and Technology Yangzhou University Yangzhou China; ^6^ Tasmanian Institute of Agriculture University of Tasmania Sandy Bay Tasmania Australia; ^7^ Laboratory of Animal Food Function, Graduate School of Agricultural Science Tohoku University Sendai Japan; ^8^ Laboratory of Nutrition, Graduate School of Agricultural Science Tohoku University Sendai Japan; ^9^ The Cattle Museum, Maesawa Oshu Japan

**Keywords:** *Bacillus subtilis* C‐3102 strain, bovine mastitis, dairy cattle, probiotics, prophylactic effect

## Abstract

Mastitis is a very common inflammatory disease of the mammary gland of dairy cows, resulting in a reduction of milk production and quality. Probiotics may serve as an alternative to antibiotics to prevent mastitis, and the use of probiotics in this way may lessen the risk of antibiotic resistant bacteria developing. We investigated the effect of oral feeding of probiotic *Bacillus subtilis* (BS) C‐3102 strain on the onset of mastitis in dairy cows with a previous history of mastitis. BS feeding significantly decreased the incidence of mastitis, the average number of medication days and the average number of days when milk was discarded, and maintained the mean SCC in milk at a level substantially lower than the control group. BS feeding was associated with lower levels of cortisol and TBARS and increased the proportion of CD4^+^ T cells and CD11c^+^ CD172a^high^ dendritic cells in the blood by flow cytometry analysis. Parturition increased the migrating frequency of granulocytes toward a milk chemoattractant cyclophilin A in the control cows, however, this was reduced by BS feeding, possibly indicating a decreased sensitivity of peripheral granulocytes to cyclophilin A. These results reveal that *B. subtilis* C‐3102 has potential as a probiotic and has preventative capacity against mastitis in dairy cows.

## 1 INTRODUCTION

Mastitis is an inflammatory disorder occurring in the mammary gland and is one of the most frequently occurring and costly diseases in dairy cows. Mastitis is a major problem in the dairy industry because it causes huge economic loss due to a reduction of milk production and a decrease of milk quality (Andersen et al., 2011; Rajala‐Schultz et al., 1999). A frustrating aspect of mastitis is its recurrent nature. Recurrence rates of clinical mastitis are approximately 50%, causing an increase in culling rates and reducing the longevity of the animals in the herd (Bar et al., 2008; Wente et al., 2020). Mastitis is caused by milking stress or multiple pathogenic microorganisms, such as *Streptococcus agalactiae*, *Staphylococcus aureus*, *Streptococcus* spp., coliforms *Escherichia coli*, *Klebsiella* spp. and *Mycoplasma* spp. (Oliveira et al., 2013; Ruegg, 2017). These bacteria are especially important, because they often infect the mammary gland shortly after drying off and before parturition, when immunosuppression of cows increases the incidence of mastitis compared with the incidence during lactation (Sordillo, 2005).

Some vaccines have been licensed in several countries to prevent the onset of mastitis (Tashakkori et al., 2020). A limitation of the vaccines is that the targets depend on their composition, such as *S. aureus* and *E. coli*, despite numerous pathogens being known to cause mastitis. The most common treatment method available against bovine mastitis is intra‐mammary infusion of antibiotics. Although antimicrobial therapy is not required in all cases of clinical mastitis, antibiotics have been used extensively for the control of bacterial infections in veterinary medicine. However, the excessive use of antibiotics raises the risk of generating antibiotic‐resistant bacteria, limiting their future efficiency to treat disease. Therefore, there is a need to explore alternative approaches for the treatment of mastitis.

Probiotic bacteria may be used as an alternative to antibiotics. The term “probiotic” is used to mean beneficial microorganisms for humans and animals, to improve the microbial environment in the gut. Several microorganisms, such as *Lactobacillus* and *Bifidobacterium*, are well known as probiotics (Suez et al., 2019). However, compared with humans and mice, the development of probiotics for livestock often requires further studies to confirm that the administered animals can be protected from disease, such as mastitis in dairy cattle.

*Bacillus subtilis* (BS) forms spores possessing high resistance to harsh conditions. After the administration of BS, digestive enzymes, such as pepsin in the stomach, facilitate the germination of its spores, resulting in an elevation of oxygen consumption and a subsequent increase of anaerobic conditions in the gut. As a result, numerous beneficial microorganisms grow well thereafter. *B. subtilis* C‐3102 strain (BS C‐3102) has been extensively used worldwide as a probiotic feed additive for livestock since 1986 (Jeong & Kim, 2014). Oral feeding with BS C‐3102 increases the amount of feed consumption throughout the reproductive cycle in pigs, resulting not only in a reduction in the loss of body weight especially at the end of lactation but also in the significant increase of weaning weight in the offspring. A report on intestinal microflora has confirmed that the frequencies of unbeneficial bacteria (e.g., *E. coli* and *Clostridium*) decrease in the gut of lactating sows and piglets after oral feeding with BS C‐3102 (Kritas et al., 2015). BS C‐3102 has also been shown to be a useful probiotic feed for chickens, as it increases the frequency of beneficial *Lactobacillus* and decreases the proportions of *E. coli* and *Salmonella* in the gut of chickens (Jeong & Kim, 2014). However, so far, little research has been conducted to address the effect of BS C‐3102 on dairy farming and milk production‐associated diseases. In order to apply BS C‐3102 as a potential probiotic for dairy cattle, we investigated the effect of oral feeding with BS C‐3102 on the onset of mastitis in dairy cows with a previous history of mastitis.

## 2 MATERIALS AND METHODS

### 2.1 Animals

Holstein cows at Miyagi Prefectural Livestock Experiment Station were used. All animal handing and experimental protocols were conducted in compliance with guidance approved by the Tohoku University Environmental and Safety Committee on Experimental Animal Care and Use (approval number: 2013AgA‐043 and 2016AgA‐018). Twenty‐six multiparous cows and 27 heifers were used in this study.

### 2.2 Experimental design with BS C‐3102

The BS C‐3102 strain used in this study was purchased from Asahi Biocycle Co., Ltd. (Tokyo, Japan). Holstein dairy cattle had a history of developing mastitis during the previous lactation periods and were divided into a control group and a BS‐feeding group. In addition, healthy heifers were separated into a control group (*n* = 11) and experimental group (*n* = 12) for the experiments on somatic cell count (SCC). The cattle were fed a blend of commercial ration for dry cows and commercial concentrate twice daily (09:30 and 16:30) to meet 110% of their energy and protein requirements during the 3 weeks prior to parturition. Thereafter, throughout the lactation period, they were fed a ration including orchard grass‐based grass silage and corn silage and a commercial concentrate twice daily (09:30 and 16:30) to meet 100% of their energy and protein requirements. All trial diets were formulated to meet the Japanese Feeding Standard for dairy Cattle (National Agriculture and Food Research Organization, 2006).

To address the effects of feeding with BS on the incidence of mastitis and on the hematological and immunological changes, the BS‐feeding group was fed twice a day with a regular diet based on grass and corn silage together with 20 g of BS C‐3102 (3.0 × 10^9^ cfu/cow) for 11 months from almost 1 month before parturition until the end of the lactation period. The control group was fed the regular diet without any supplementation. The incidence of mastitis, the periods of medical treatment with antibiotics, and the number of days when milk was discarded after medical care were recorded for 3 months after parturition. Milk samples were collected twice a day and were tested with the California Mastitis Test (CMT) with PL tester (Nippon Zenyaku Kogyo Co., Ltd., Koriyama, Fukushima, Japan) to determine mastitis incidence. The health of the mammary gland was confirmed every day by physical examination. In addition, SCC in milk was determined precisely using a Fossomatic Minor automated somatic cell counter (Foss A/S, Hillerød, Denmark).

### 2.3 Plasma analyses

Peripheral blood was collected from the cows, before feeding and after milking (09:30), once a month on 12 occasions over the course of the study using a heparinized vacutainer tube, and the concentrations of glucose, blood urea nitrogen (BUN), non‐esterified fatty acid (NEFA), total cholesterol (T‐chol), cortisol and thiobarbituric acid reactive substances (TBARS) were measured. Following commercial kits were used according to the manufacturers' instructions: Glucose C2 (Wako Pure Chemical Corporation, Osaka, Japan), either Urea N (Wako) or SEIKEN UN‐S (Denka Co., Ltd., Tokyo, Japan), NEFA C (Wako), Cholesterol E (Wako), Cortisol EIA kit (Enzo Life Sciences, Inc., New York, USA) and TBARS Assay Kit (Cayman Chemical Company, Michigan, USA).

### 2.4 Flow cytometry analyses

Jugular venous blood (100 ml) was obtained from the cows on 12 occasions, once a month over the course of the study, into tubes containing sodium heparin. For the determination of the population of leucocytes, peripheral blood was treated with 0.83% ammonium chloride to lyse erythrocytes. After centrifugation, cells, which were mostly leucocytes, were subjected to flow cytometry analyses using primary monoclonal antibodies and fluorescence‐conjugated secondary antibodies as shown in Tables 1 and 2. To determine the frequency of granulocytes, monocytes, T cells, and B cells, the leucocytes were stained with either anti‐granulocyte (CH138A), anti‐CD14 (CAM36A), anti‐CD3 (MM1A) or anti‐CD21 (GB25A) antibody for 30 min at 4°C after blocking with goat or rat IgG (Sigma‐Aldrich Co. LLC, Missouri, USA). After washing, the cells were treated with Alexa Fluor 488‐ or PE‐conjugated corresponding secondary antibodies for 30 min at 4°C. To further determine the subset of T cells, peripheral blood mononuclear cells (PBMC) were isolated from the buffy coat by density gradient centrifugation at 600 g for 30 min at 18°C using Lympholyte‐H (1.077 g/ml, Cedarlane, Burlington, Ontario, Canada). PBMC were stained with monoclonal antibodies specific for CD4 (ILA11A), CD8 (BAQ111A), γδTCR1‐N24 (GB21A) or WC1‐N1 (B7A1) followed by FITC‐ or PE‐conjugated secondary antibodies.

**TABLE 1:** Primary mouse monoclonal antibodies^a^

Bovine antigen	Clone	Isotype	Working conc. (μg/ml)
Granulocytes	CH138A	IgM	0.8
CD14 (monocytes)	CAM36A	IgG1	20.0
CD3 (T cells)	MM1A	IgG1	1.0
CD21 (B cells)	GB25A	IgG1	20.0
B cells	BAQ44A	IgM	20.0
CD4 (T cells)	ILA11A	IgG2a	7.0
CD8 (T cells)	BAQ111A	IgM	0.1
γδTCR1‐N24 (T cells)	GB21A	IgG2b	1.3
WC1‐N1 (T cells)	B7A1	IgM	0.1
CD11c (monocytes, B cells, T cells, DCs)	BAQ153A	IgM	0.2
CD172a (monocytes, DCs)	DH59B	IgG1	10.0
CD25 (IL‐2R)	CACT116A	IgG1	7.0

^a^ All mouse monoclonal antibodies against bovine blood cells were purchased from Washington State University Monoclonal Antibody Center.

**TABLE 2:** Secondary antibodies

Antibody	Conjugation	Dilution
Rat anti‐mouse IgG1 monoclonal^a^	PerCP	1:10
Goat anti‐mouse IgG(H+L) polyclonal^b^	Alexa Fluor 488	1:250
Goat anti‐mouse IgG2a polyclonal^c^	FITC	1:75
Goat anti‐mouse IgG2b polyclonal^c^	FITC	1:75
Goat anti‐mouse IgM polyclonal^c^	PE	1:50
Rat anti‐mouse IgG1 monoclonal^d^	MicroBeads	1:5
Rat anti‐mouse IgM monoclonal^d^	MicroBeads	1:5

^a^ BD biosciences.

^b^ Invitrogen.

^c^ Southern Biotech.

^d^ Miltenyi Biotech.

To detect dendritic cells (DCs), a negative selection was first performed to remove T cells, B cells, and monocytes from the PBMC according to methods noted in previous studies (Miyazawa et al., 2006; Zhuang et al., 2018). After blocking with control goat and rat IgG, PBMC were stained with a mixture of anti‐CD3 (MM1A), anti‐B cells (BAQ44A) and anti‐CD14 (CAM36A) antibodies for 30 min at 4°C, followed by an incubation with microbeads‐conjugated secondary antibodies. Non‐T, non‐B, and non‐monocyte cells containing DCs were negatively selected using autoMACS (Miltenyi Biotec, Bergisch Gladbach, Germany). The enriched cells were then stained with anti‐CD11c (BAQ153A) and anti‐CD172a (DH59B) antibodies followed by PE‐ and PerCP‐conjugated secondary antibodies. Flow cytometry data were determined using either FACSCalibur (BD Biosciences, New Jersey, USA) or BD Accuri C6 Flow Cytometer (BD Biosciences). In each experiment, cells incubated with isotype‐matched antibodies and fluorescent antibodies were selected as controls.

### 2.5 Chemotaxis analysis

Firstly, peripheral blood samples were obtained from 15 clinically healthy Holstein cows into heparinized vacutainer tubes at 1 month before parturition. The cows were either from a control feeding group (*n* = 5) or the BS‐feeding group (*n* = 10). The control group was fed a normal diet, and the BS‐feeding group was fed twice a day a normal diet together with 20 g of BS C‐3102 (3 × 10^9^ cfu/cow) for 2 months, 1 month before until 1 month after parturition. Secondly, peripheral blood samples were obtained from the cows at 1 month after parturition. Granulocytes in the peripheral blood were used for a chemotaxis analysis with recombinant bovine cyclophilin A (rbCyPA), as described previously (Takanashi et al., 2015). Briefly, PBMC were removed by centrifugation with Lympholyte‐H, and erythrocytes were lysed with 0.83% (w/v) of ammonium chloride to obtain the granulocytes. The chemotaxis analysis assay was performed using Transwell® inserts for 24 well plate with 5.0 μm pore polycarbonate membrane (Corning Inc., New York, USA). Granulocytes were suspended in STEMPRO® (Thermo Fisher Scientific Inc., Waltham, Massachusetts, USA) and seeded into the upper chambers of the inserts at a cell density of 1.0 × 10^6^ cells/cm^2^/100 μL. The lower chambers were filled with or without 100 ng/ml of rbCyPA. After incubation for 90 min at 37°C in a 5% CO_2_ incubator, the membranes of the Transwell® inserts were stained with Giemsa regent (Nacalai Tesque, Inc., Kyoto, Japan). Five images (76,800 μm^2^/image) were randomly obtained from 3 or 4 membranes using an AX70 (Olympus Corporation, Tokyo, Japan) Microscope. The numbers of migrating granulocytes were counted in the lower side of the insert membrane.

### 2.6 Statistical analysis

Statistical analyses were performed using a statistical software jSTAT and Prism 7 (GraphPad). Data sets were tested for normal distribution. Student *t* test was performed for the data in Figure 7. As all other data were not distributed normally, the nonparametric Mann–Whitney *U* test was used.

## 3 RESULTS

### 3.1 Prophylactic effect of BS feeding on the incidence of mastitis in dairy cows

**FIGURE 1:**
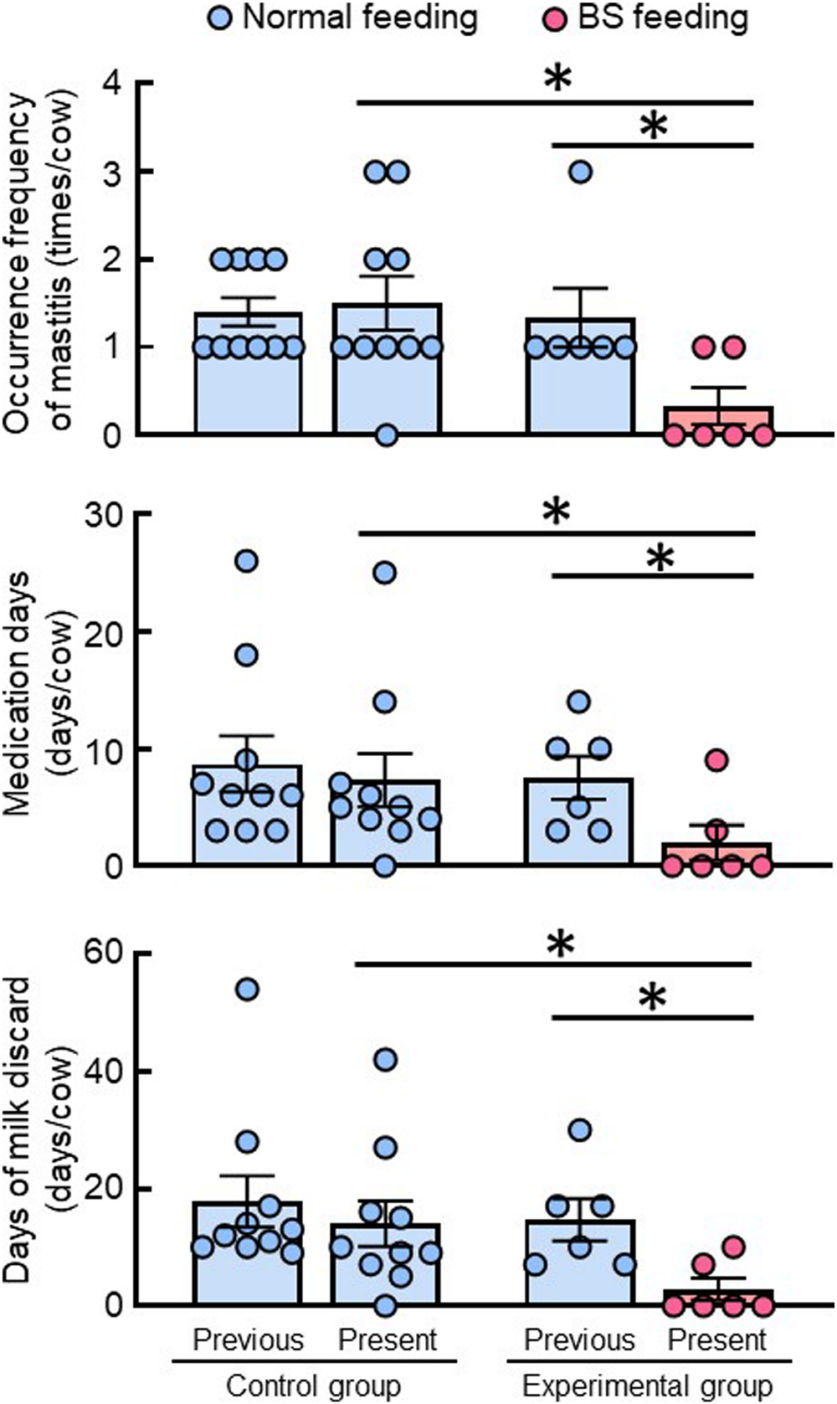
Prophylactic effect of *Bacillus subtilis* (BS) feeding on mastitis in dairy cows. All dairy cows had a history of mastitis. The control group (*n* = 10, calving number = 4.1 ± 1.7 [mean ± S. D.]) was fed a normal diet. The experimental group (*n* = 6, calving number = 4.5 ± 1.0) was fed a BS C‐3102 in their diet between 1 month before parturition and the end of the lactation period. Blue or red bars represent the mean of each group with standard errors. *P < 0.05.

We investigated the prophylactic effect of BS C‐3102 on the incidence of mastitis in dairy cows. The control group was fed a normal diet, and the experimental group was fed a diet containing BS from 1 month before parturition to the end of the lactation period. This experiment focused on 3 months after parturition and made a comparison between the control and treatment groups. All dairy cows had encountered mastitis in the previous lactation, though there were no differences in the occurrence frequency of mastitis, the average number of medication days or the number of days that the milk was discarded in the previous lactation between the control group and the experimental group (Figure 1). In the current lactation, the control cows developed mastitis at the same frequency as the previous lactation. In contrast, BS feeding significantly decreased the incidence of mastitis, the average number of medication days and the number of days that the milk was discarded, compared with the values of the previous lactation.

**FIGURE 2:**
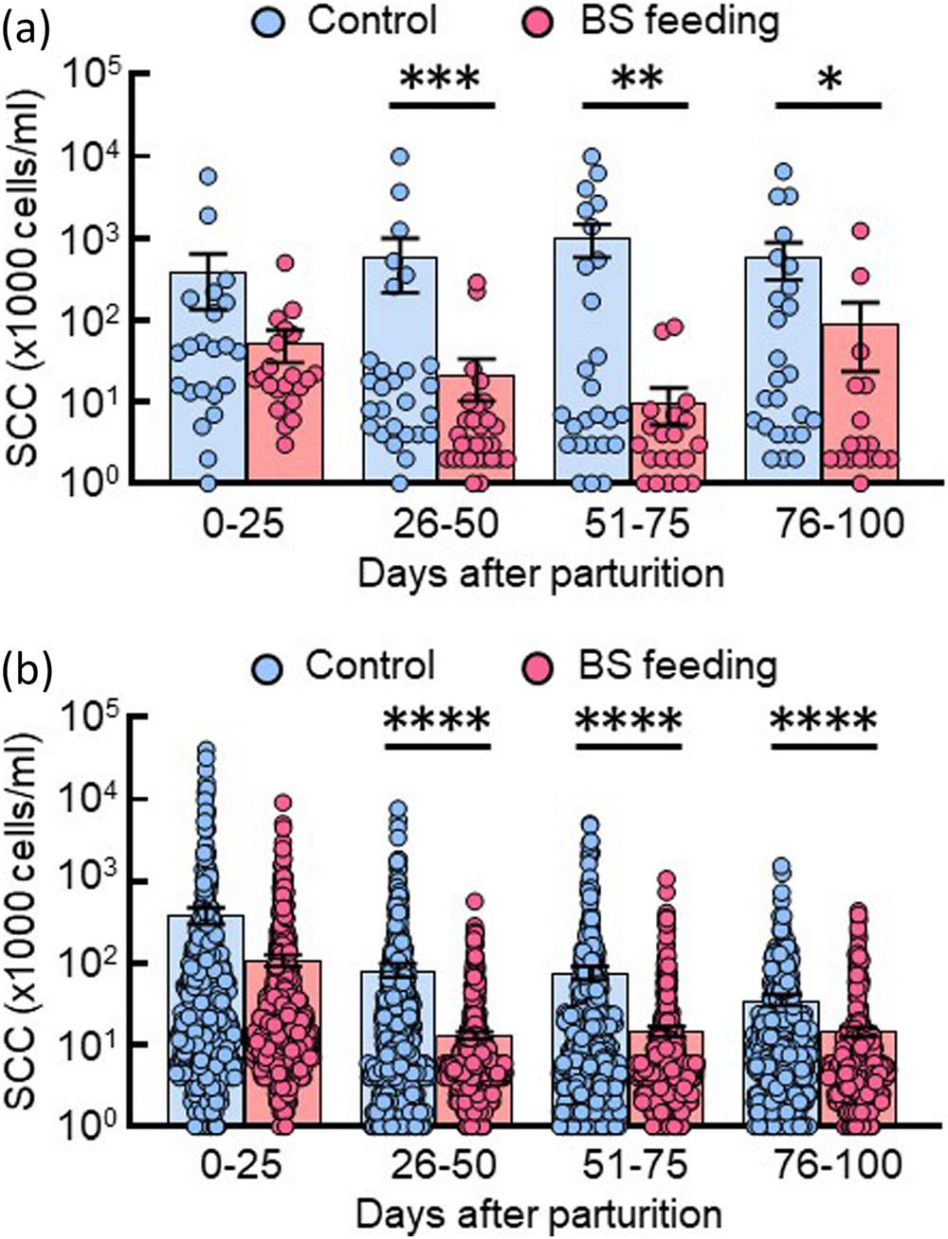
Effect of *Bacillus subtilis* (BS) feeding on somatic cell count (SCC) in milk of dairy cows. Cows with a history of mastitis were divided into the control group (*n* = 6, blue circle) and the experimental group (*n* = 6, red circle) (a). Heifers were separated into the control group (*n* = 11, blue circle) and the experimental group (*n* = 12, red circle) (b). The control group was fed a normal diet, and the experimental group was fed the BS diet from 1 month before parturition. Milk samples were collected for 100 days after parturition, and their SCC was determined. These data were divided into four periods of 25 days each over the 100 days. The values below the lower limit (1000 cells/ml) are not shown in the figures. Blue or red bars represent the mean of SCC with standard errors. *P < 0.05, **P < 0.01, ***P < 0.001, ****P < 0.0001.

Results from milk samples were reported as the median for the periods 1–25, 26–50, 51–75 and 76–100 days postpartum. The mean SCC in milk of the control group continued to be higher than 300,000 cells/ml (Figure 2a). There was no difference in the mean SCC in milk at 0–25 days after parturition between the control group and the BS‐feeding group. This period includes the period when the colostrum is secreted, and the SCC is generally high because various components such as mammary epithelial cells and leukocytes are present in the colostrum in high concentrations (Valckenier et al., 2020). However, the mean SCC in milk of the BS‐feeding group was substantially lower thereafter and reached an average of 10,000 cells/ml at 51 to 75 days after parturition. After 26 days of parturition, there was a significant difference in the mean SCC in milk between the control group and the BS‐feeding group (Figure 2a). To confirm whether BS feeding maintains a low level of SCC in milk, we conducted a similar study using healthy heifers. SCC in the BS‐feeding group was significantly lower after 26 days of parturition than those in the control cows, as for the results of cows with a previous history of mastitis (Figure 2b). These data reveal that the BS C‐3102 has a prophylactic ability against the incidence of mastitis in dairy cows.

### 3.2 Effect of BS feeding on concentrations of plasma metabolites

**FIGURE 3:**
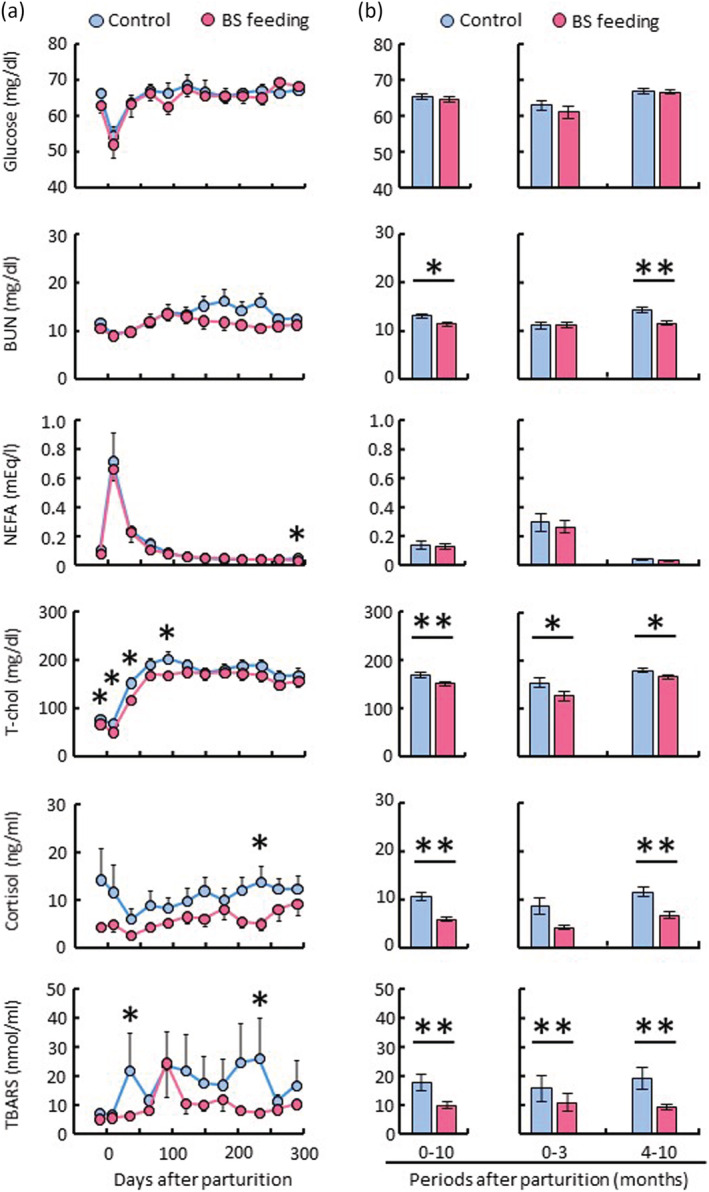
Effect of *Bacillus subtilis* (BS) feeding on the concentrations of plasma metabolites. The control group (*n* = 10) was fed a normal diet, and the experimental group (*n* = 10) was fed a BS diet between 1 month before parturition and the end of the lactation. Blood samples were obtained on 12 occasions, once a month from the dairy cows. The concentrations of glucose, urea nitrogen (BUN), non‐esterified fatty acid (NEFA), total cholesterol (T‐chol), cortisol and thiobarbituric acid reactive substances (TBARS) in plasma were measured (a). The comparison analysis shows the differences in the averages of plasma metabolites between the control group and the BS‐feeding group in the total lactation period, the early and the mid and late stages of lactation (b). *P < 0.05, **P < 0.01.

To clarify the mechanism of the prophylactic effect of BS C‐3102 on mastitis onset, we focused on metabolic function and stress indicators. Glucose, BUN, NEFA, T‐chol, cortisol, and TBARS were measured (Figure 3). Plasma glucose concentrations in both control and BS‐feeding cows immediately decreased after parturition and recovered to normal levels 1 month later. The plasma BUN concentrations of the control cows immediately decreased after parturition, then gradually increased for 90 days, continued to be at the high level but then decreased after 270 days. In contrast, the plasma BUN concentration of the BS‐feeding cows decreased after 150 days from parturition. There were significant differences in the average of BUN concentration over the total lactation period and during the mid and late stages of lactation between the groups. Plasma NEFA concentrations of both control and BS‐feeding cows immediately increased after parturition and returned to the previous levels 1 or 2 months later. Plasma T‐chol concentrations of both control and BS‐feeding cows immediately decreased after parturition and gradually increased for 3 months. However, there was a significant difference in the average plasma T‐chol concentrations between the control group and the BS‐feeding group. Plasma concentrations of cortisol and TBARS were higher in the control group than in the BS‐feeding group. These data suggest that BS feeding is associated with a faster recovery of plasma metabolites after parturition.

### 3.3 Effect of BS feeding on proportion of peripheral leucocytes during lactation

**FIGURE 4:**
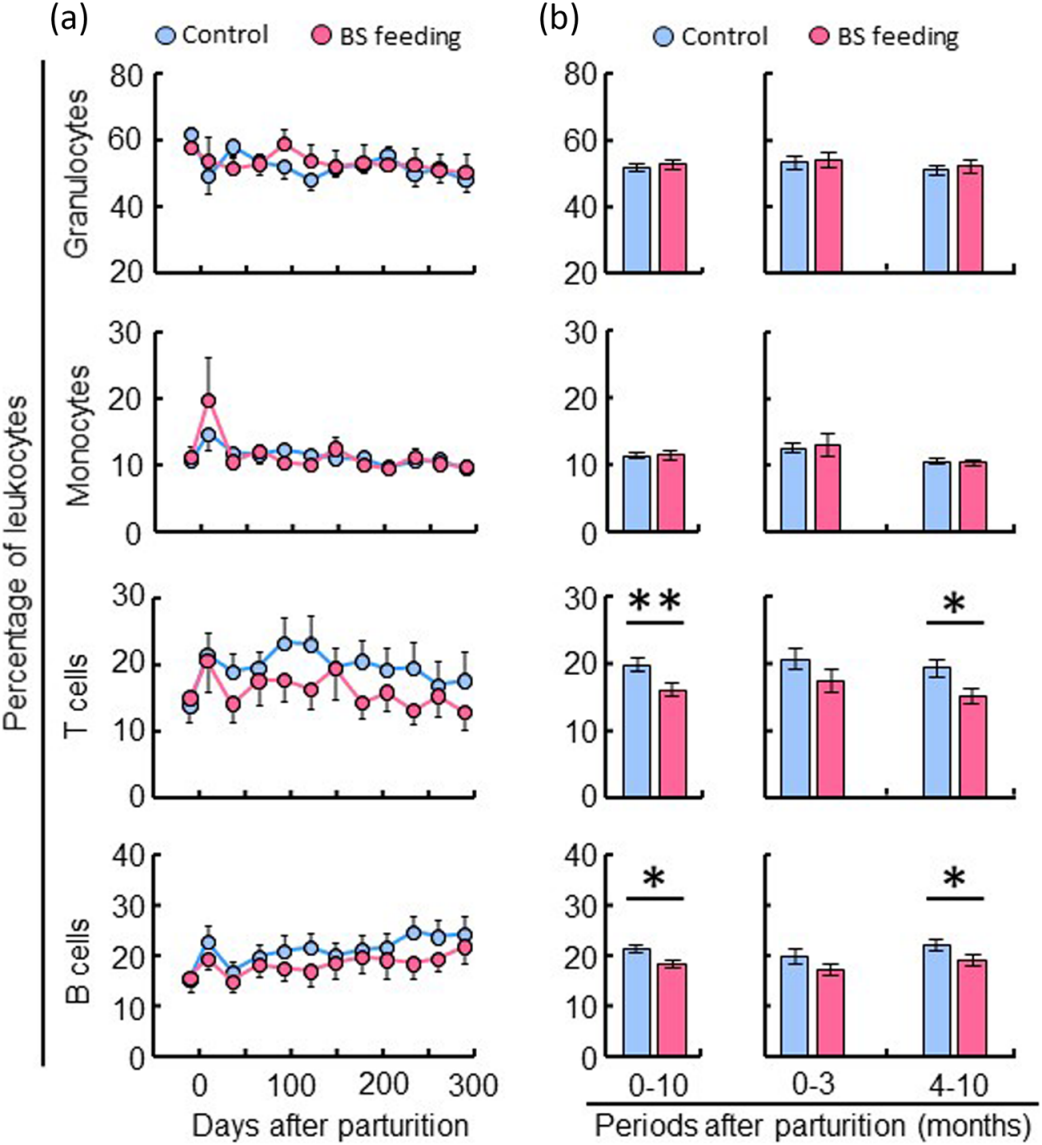
Effect of *Bacillus subtilis* (BS) feeding on the proportion of peripheral leucocytes during lactation. The control group (*n* = 10) was fed a normal diet, and the experimental group (*n* = 10) was fed a BS diet between 1 month before parturition and the end of the lactation. Peripheral blood was obtained on 12 occasions, once a month from the dairy cows. The percentages of granulocytes, monocytes, T cells, and B cells were analyzed in peripheral blood by flow cytometry (a). The comparison analysis shows the differences in the averages of peripheral leucocytes between the control group and the BS‐feeding group in the total lactation period, the early and the mid and late stages of lactation (b). *P < 0.05, **P < 0.01.

To investigate the changes in immune function in the mammary gland caused by BS C‐3102, the proportions of various immune cells were analyzed. Over the 12 months of the study, there were no statistical differences in the percentages of granulocytes, monocytes, T cells, and B cells in peripheral blood between the control and BS‐feeding group (Figure 4a). In contrast, the averages of T cells and B cells were lower in the BS‐feeding group than in the control group during the total lactation period and the mid and late stages of lactation (Figure 4b).

**FIGURE 5:**
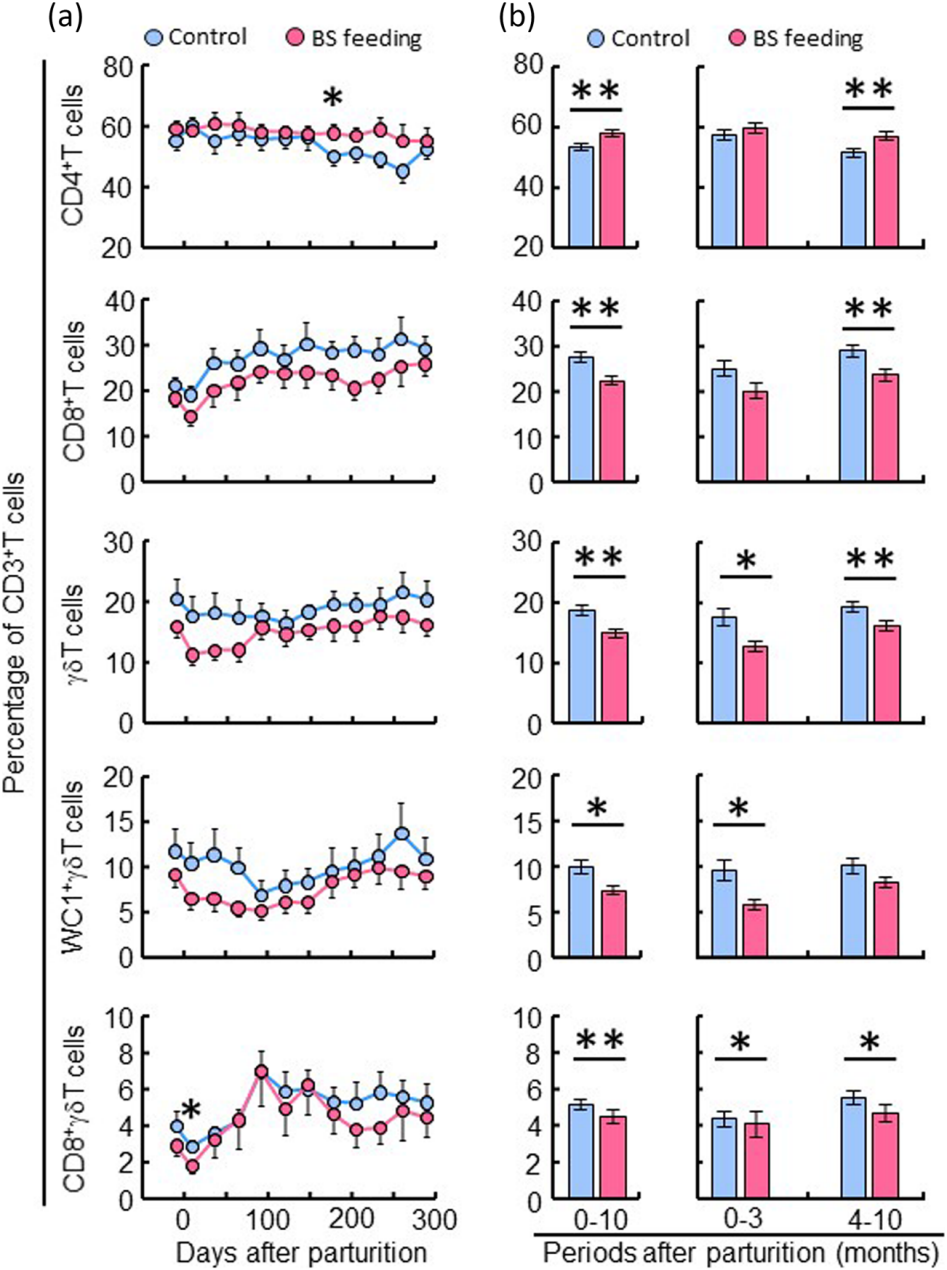
Effects *Bacillus subtilis* (BS) feeding on the proportion of peripheral CD3^+^ T cells during lactation. During the present lactation, the control group (*n* = 10) was fed a normal diet, and the experimental group (*n* = 10) was fed a BS diet between 1 month before parturition and the end of the lactation. Peripheral blood was obtained from the dairy cows on 12 occasions from just before parturition to the end of lactation. The percentages of CD4^+^ T cells, CD8^+^ T cells, γδ^+^ T cells, WC1^+^ γδ^+^ T cells and CD8^+^ γδ^+^ T cells were analyzed in CD3^+^ T cells by flow cytometry (a). The comparison analysis shows the differences in CD3^+^ T cells between the control group and the BS‐feeding group in the total lactation period, the early and the late stages of lactation, respectively (b). *P < 0.05, **P < 0.01.

The proportion in the CD4^+^ T cells of BS‐feeding cows was maintained at 60% in CD3^+^ T cells, however, that of the control cows decreased during the late lactation period. The proportions of CD8^+^ T cells, γδ^+^ T cells, WC1^+^ γδ^+^ T cells and CD8^+^ γδ^+^ T cells, which were associated with inflammation, were either significantly lower or tended to be lower in the BS‐treated group (Figure 5a). There were significant differences in the averages of CD8^+^ T cells, γδ^+^ T cells, WC1^+^ γδ^+^ T cells and CD8^+^ γδ^+^ T cells at the total lactation period between control and BS‐feeding group (Figure 5b). These data suggest that BS feeding decreased the proportion of the inflammatory T cell population via their prophylactic ability against the incidence of mastitis in dairy cows.

### 3.4 Influence of supplementation with BS on proportion of peripheral DCs

**FIGURE 6:**
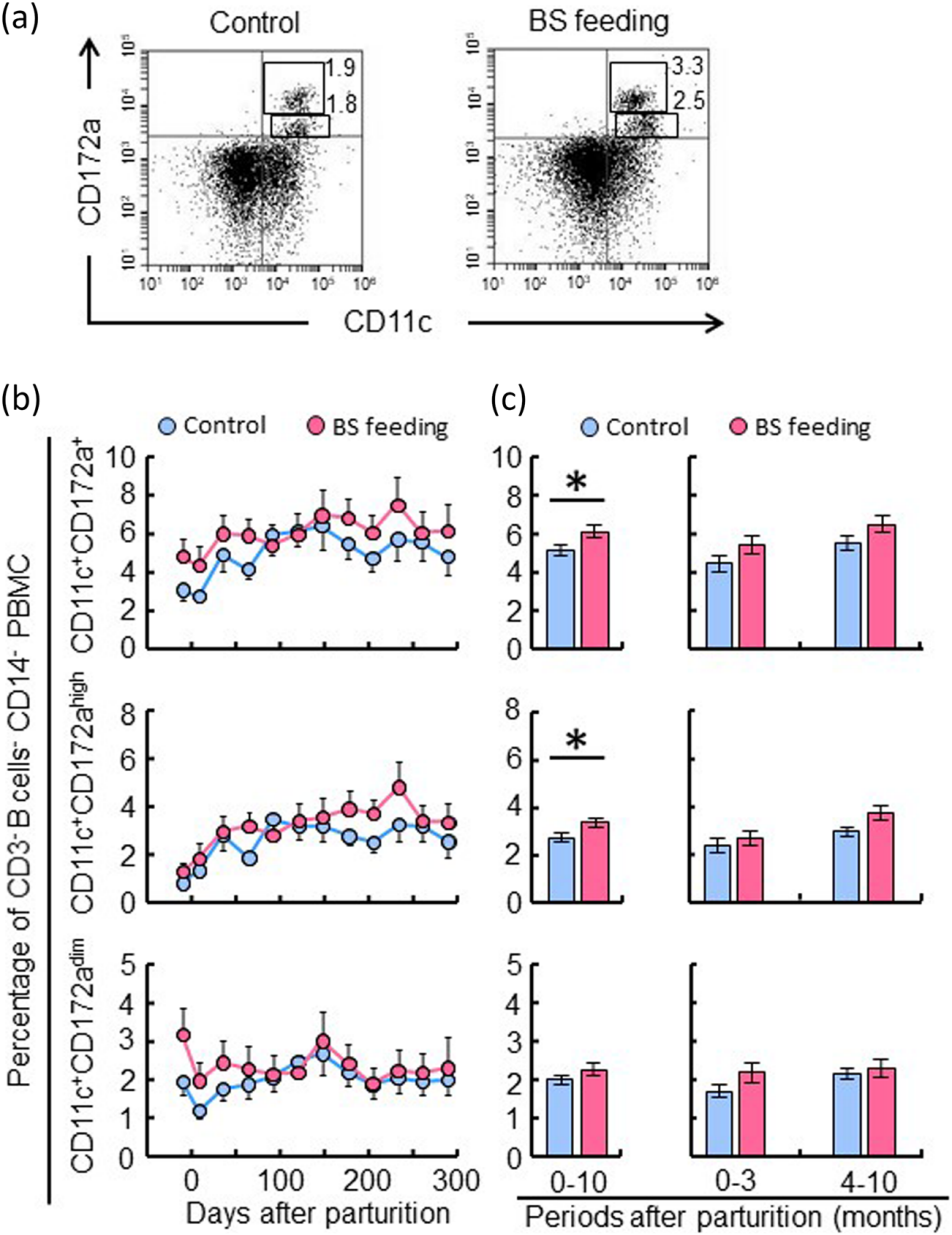
Effect of *Bacillus subtilis* (BS) feeding on peripheral dendritic cells during lactation. During the present lactation period, the control group (*n* = 10) was fed a normal diet, and the experimental group (*n* = 10) was fed a BS diet between 1 month before parturition and the end of the lactation. Peripheral blood was obtained from dairy cows on 12 occasions from just before parturition to the end of lactation. T cells, B cells, and monocytes were removed from peripheral blood mononuclear cell (PBMC) by the negative selection using magnetic‐activated cell sorting (MACS) with anti‐CD3 (MM1A), anti‐B cells (BAQ44A), and anti‐CD14 (CAM36A) antibodies. After the negative selection, the expression of the surface molecules CD172a and CD11c was analyzed on the negative‐selected cells by flow cytometry (a). The percentages of CD11c^+^ CD172a^+^, CD11c^+^ CD172a^high^ and CD11c^+^ CD172a^dim^ dendritic cells were counted during the lactation (b). The comparison analysis shows the differences in peripheral dendritic cells between the control group and the BS‐feeding group in the total lactation period, the early and the late stages of lactation, respectively (c). *P < 0.05.

A subset of the bovine peripheral blood DCs was identified as CD172a^+^/CD11c^+^/MHC class II^+^ cells in the CD3^−^/B‐B2^−^/CD14^−^ population (Miyazawa et al., 2006; Zhuang et al., 2018). However, it is well‐known that CD11c is highly expressed on monocytes, macrophages (Mø) and natural killer (NK) cells, and that CD172a^+^/CD11c^+^ cells possibly include a subset of T cells, B cells, NK cells and monocytes/Mø. Next, we attempted to remove these cell populations from PBMC using specific monoclonal antibodies for each by a magnetic‐activated cell sorting (MACS) system. Therefore, CD172a^+^/CD11c^+^ cells in the negative‐selected cells were considered as bovine peripheral blood DCs. CD172a^+^/CD11c^+^ DCs were found to represent 3.7% in the control‐feeding group and 5.8% in the BS‐feeding group (Figure 6a). In addition, bovine CD172a^+^/CD11c^+^ DCs were classified into two populations of CD11c^+^ CD172a^high^ and CD11c^+^ CD172a^dim^ according to the intensity of CD172a (Figure 6a). Throughout the analysis period, there were no differences in the proportion of CD172a^+^/CD11c^+^ and CD11c^+^ CD172a^high^ or CD11c^+^ CD172a^dim^ DCs between the control‐ and the BS‐feeding groups (Figure 6b). However, the average of CD172a^+^/CD11c^+^ DCs was significantly higher in the BS‐feeding group than those in the control group during lactation period, which depended on the increase of CD11c^+^ CD172a^high^ DCs in the BS‐feeding group (Figure 6c).

### 3.5 Effect of BS feeding on chemotactic activity of peripheral granulocytes

**FIGURE 7:**
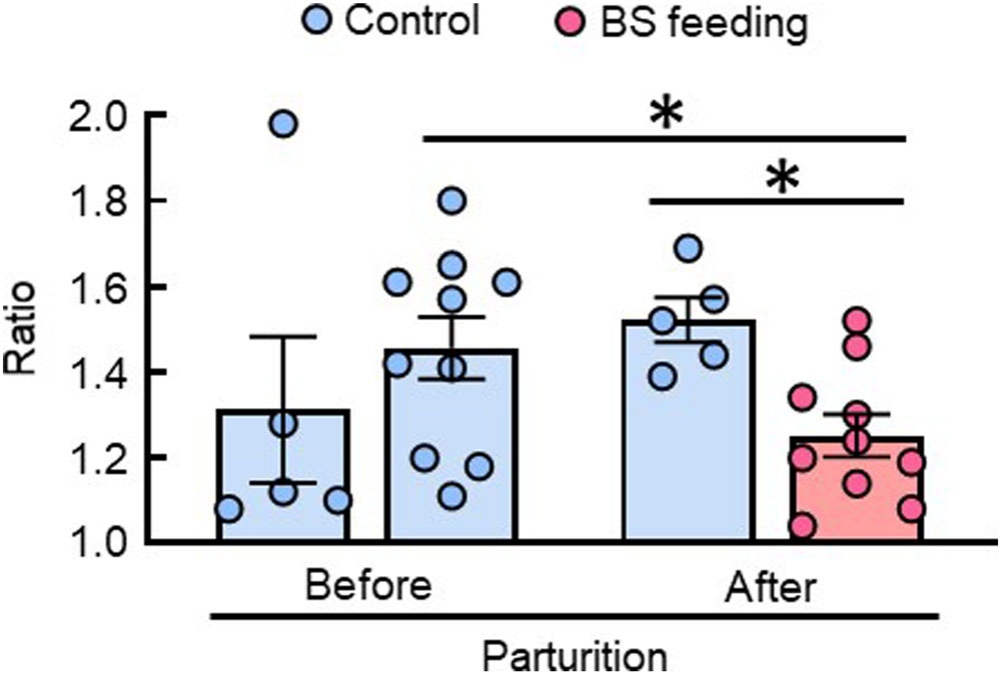
Effect of *Bacillus subtilis* (BS) feeding on chemotactic activity of peripheral granulocytes. Blood samples were obtained from dairy cows at 1 month before parturition. Then, the control group (*n* = 5) was fed a normal diet, and the experimental group (*n* = 10) was fed a BS diet from 1 month before parturition. At 1 month after parturition, second blood samples were obtained from dairy cows. Granulocytes were purified by centrifugation with Lympholyte‐H. Chemotactic analysis with 100 ng/ml of rbCyPA was performed using Transwell® inserts. After incubations for 90 min, the membranes were stained with Giemsa reagent. The migrating granulocytes were counted using a microscope. The ratio of the cell number of migrating granulocytes with rbCyPA to that without it is indicated. Results are reported as mean ± SEM. *P < 0.05.

We have previously reported that CyPA was secreted from bovine mammary epithelial cells and that CyPA possesses chemotactic activity to recruit inflammatory cells (e.g., granulocytes) in cattle (Takanashi et al., 2015). Therefore, we enriched granulocytes from peripheral blood cells of dairy cows. The granulocytes were cultured in the upper chambers of Transwell® inserts and stimulated with 100 ng/ml of rbCyPA in the lower chambers. The average migration ratio before parturition was 1.3 and 1.5 in the normal‐feeding and the BS‐feeding cows, respectively (Figure 7). At 1 month after parturition, the granulocytes were subjected to a chemotactic assay. Feeding of BS significantly reduced the migrating frequencies of peripheral granulocytes toward CyPA. In addition, there was a significant difference in the average of migration ratio between the normal‐feeding and the BS‐feeding groups after parturition. These data suggest that BS feeding may decrease the sensitivity of peripheral granulocytes to CyPA.

## 4 DISCUSSION

The milk yield of dairy cattle peaks at about 3 months after parturition. The period up to this time is when most health problems occur, including most incidences of mastitis (Andersen et al., 2011). If mastitis develops during this period, the total milk yield will not reach the pre‐mastitis potential (Rajala‐Schultz et al., 1999). Indeed, the onset of mastitis before the peak of lactation has a large impact on subsequent milk yield and is associated with much higher rates of culling (Seegers et al., 2003). In addition, the occurrence of intramammary infection during the dry period increases the risk of infection during the subsequent lactation period (Green et al., 2002). Possible reasons for the onset of mastitis during this period include the reduction of resistance to bacterial exposure by immunosuppression and the increased supply of milk nutrients for bacterial growth in the perinatal period, the stress of approaching parturition, and the negative energy balance associated with parturition (Sordillo, 2005). It has also been reported that infection during the dry period and subsequent lactation might alter the mammary gland environment, and that previous infection may impair innate defense mechanisms (Green et al., 2002). In this study, we investigated the prophylactic effect of the BS C‐3102 on the incidence of mastitis in dairy cows with a previous history of mastitis. This experiment focused on the dry period and 3 months after parturition, while BS feeding started at 1 month before parturition. BS feeding significantly decreased the incidence of mastitis, compared with that of the previous lactation and compared with the control cows (Figure 1). In addition, the BS C‐3102 kept the SCC in milk at a low level (Figure 2). Several microorganisms have been evaluated for their probiotic activity. Probiotics are becoming more common in the treatment of several inflammatory diseases. Lactic acid bacteria are the major group of probiotic organisms (Dhama et al., 2017). Lactic acid bacteria can provide protection against mastitis when used as feed supplements, teat dips, and intramammary inoculations (Sharun et al., 2021). Here, we report for the first time that the BS C‐3102 strain can significantly reduce the risk of the development of mastitis in dairy cows with a previous history of mastitis.

The homeostatic balance in postpartum dairy cows is disrupted by the redirection of nutrients toward the mammary gland in support of milk production. The high metabolic priority for milk production, accompanied by limited feed intake around parturition, results in a high degree of body fat mobilization. Under these conditions, the metabolic fuel source of many peripheral organs is switched from carbohydrate to fat utilization to spare glucose for milk production and to ensure partitioning of tissue‐ and dietary‐derived nutrients toward the mammary gland (Bauman & Bruce Currie, 1980; Kuhla et al., 2016). It is well‐known that plasma metabolite concentrations in dairy cows fluctuate significantly before and after parturition, such as plasma glucose, NEFA (Chandra et al., 2013; Dyck et al., 2011; Laeger et al., 2013; Salin et al., 2018), BUN (Vallimont et al., 2001), and T‐chol (Guretzky et al., 2006; Laeger et al., 2013). During these substantial metabolic shifts, dairy cows experience metabolic or infectious disease during the transition from late gestation to early lactation (LeBlanc, 2010). In this study, there were no differences in the shape of the profiles of the metabolites in plasma between the control‐feeding group and the BS‐feeding group. Nevertheless, it should be noted that the concentrations of plasma BUN and T‐chol in the control‐feeding group increased rapidly after parturition and immediately reached the normal level, whereas the recovery in the BS‐feeding group was gradual (Figure 3). These results suggest that the BS fed group recovered their concentrations of plasma BUN and T‐chol to normal levels more smoothly, and that the BS C‐3102 may enable the avoidance of a prolonged negative energy balance duration in postpartum dairy cows.

We observed that BS feeding kept the contents of cortisol and TBARS in blood lower than the normal‐feeding group (Figure 3). As cortisol concentration is an indicator of stress, it rises in blood at the time of parturition (Chandra et al., 2018; Niederecker et al., 2018) and in tail hair from the dry period up to 90 days after parturition (Braun et al., 2017; Endo et al., 2017). TBARS is an indicator of lipid peroxidation, an increase indicating that the cows were under oxidative stress. Due to metabolic demands associated with late pregnancy, parturition, and increasing milk production in lactation, the associated increased production of reactive oxygen species (ROS) results in increased oxidative stress (Chandra et al., 2013). As recently calved dairy cows with high genetic potential and high milk yield do not have a sufficient feed intake during pregnancy, they usually have reduced milk yield relative to potential and carry an increased risk of production diseases. It has been reported that feeding *B. subtilis* *natto*, which is a strain related to BS C‐3102, increased milk yield and improved feed efficiency through a result of increased propionate in the lumen (Peng et al., 2012). Therefore, BS C‐3102 feeding also might increase feed intake efficiency and recover the supply of nutrients around parturition in dairy cows. We consider that the BS C‐3102 may have reduced the stress of the animals in such circumstances.

We analyzed various kinds of immune cell subsets in peripheral blood on 12 occasions from before parturition to the end of the lactation. The average T cell and B cell populations were lower in the BS‐feeding group than in the normal‐feeding group (Figure 4). Specifically, BS feeding increased the proportion of CD4^+^ T cells but decreased the proportions of CD8^+^ T cells, γδ T cells, WC1^+^ γδ T cells, and CD8^+^ γδ T cells (Figure 5). The previous reports show that there is a decline in the proportions of T cell in blood immune cells after parturition, such as CD8^+^ T cells (Karcher et al., 2008), γδT cells (Kimura et al., 1999, 2002), and WC1^+^ γδ T cells (Ohtsuka et al., 2004), except for CD4^+^ T cells (Karcher et al., 2008). These population changes have previously been thought to be associated with immune suppression in periparturient cows. However, Kimura et al. (2002) revealed that mastectomy eliminated many of these changes, and that the presence of the mammary gland was responsible for most of the changes of immune cell populations in periparturient dairy cows. On the other hand, it was reported that the proportion of CD8^+^ T cells was high after parturition in cows suffering from Johne's disease (Karcher et al., 2008). In this study, BS‐feeding cows had a lower percentage of CD8^+^ T cells during all lactations relative to the normally fed cows. We also observed that CD11c^+^ CD172a^+^ DCs in peripheral blood increased after parturition (Figure 6). DCs are professional antigen presenting cells specialized for antigen‐uptake and processing and play an important role in the innate and adaptive immune responses. The average number of DCs was significantly higher in the BS‐feeding group than the control group, depending on the increase of CD11c^+^ CD172a^high^ DCs. DCs regulate the population and functions of T cells during parturition (Robertson et al., 1996; Schumacher et al., 2012). DCs activated by BS feeding may migrate into the mammary gland for suppressing inflammation via the regulation of T cell functions. As mature DCs expressed co‐stimulatory molecules such as CD40, CD80 and CD86 and showed a reduced production of pro‐inflammatory cytokines (IL‐12, TNFα, IL‐6) (Lutz & Schuler, 2002), further research should explore the presence of similar phenotypes of DCs in cattle after parturition during the lactation period. Taken together, our results suggest that BS feeding may keep the proportion of T cells at normal levels, and that the T cell population changes seen in control cows may be influenced by the occurrence of mastitis.

We previously reported that CyPA was secreted from the bovine mammary epithelial cells and possessed chemotactic activity to recruit inflammatory cells (e.g., granulocytes) in cattle (Takanashi et al., 2015). We also reported that the CyPA was able to significantly increase the expression of IL1β, IL1α, CXCL8, and CXCL3 in bovine mammary epithelial cells (Islam et al., 2020). These chemokines induce the chemotaxis of granulocytes and recruit them toward the site of infection. As the content of CyPA in milk quickly increased after intermammary infection, it may be a leading indicator of elevated SCC and inflammation and mastitis. In this study, parturition increased the migrating frequency of granulocytes toward CyPA in the normally fed cows, however, BS feeding reduced it (Figure 7). These results suggest that oral feeding with BS C‐3102 might regulate undesired chronic inflammation by reducing the responsiveness of granulocytes to CyPA, reducing the level of SCC in milk.

Live probiotic bacteria (probiotics) are thought to reduce intestinal colonization by pathogens and susceptibility to infection, although the mechanisms remain poorly understood. A previous study reported that the consumption of probiotic *B. subtilis* comprehensively abolished colonization by the harmful pathogen *S. aureus* in human and murine studies and that the fengycins of *Bacillus* lipopeptides eliminates *S. aureus* by inhibiting the quorum sensing for their population density (Piewngam et al., 2018). As *S. aureus* is a major bacterium for mastitis, these findings show a probiotic‐based method for the prevention of mastitis by *S. aureus* decolonization and new ways to fight its infection. Our results reveal that BS C‐3102 is a potential probiotic with an ability to prevent mastitis in dairy cows. Therefore, it would be appropriate to experimentally show that BS C‐3102 enables resistance to infection through further studies aimed at elucidating its mechanism of action. Such an understanding would accelerate its practical application for the prevention of mastitis.

It is accepted that a large number of cows are required in order to increase the confidence that a particular bacterial strain is an effective probiotic than has been possible in this study. Clinical mastitis occurs with a high recurrence rate in cows that have had the disease previously (Bar et al., 2008; Wente et al., 2020; Zadoks et al., 2001). We have shown that the BS C‐3102 strain had a preventative capacity against mastitis in dairy cows with a previous history of mastitis. This method has some beneficial potential for the dairy industry and comes without considerable cost for use in dairy cows with a previous history of mastitis.

## CONFLICT OF INTEREST

The authors declare no conflict of interest.
